# Efficacy of antidotes (midazolam, atropine and HI-6) on nerve agent induced molecular and neuropathological changes

**DOI:** 10.1186/1471-2202-15-47

**Published:** 2014-04-04

**Authors:** Golime RamaRao, Prachiti Afley, Jyothiranjan Acharya, Bijoy Krishna Bhattacharya

**Affiliations:** 1 Biochemistry Division, Defence Research and Development Establishment, Jhansi road, Gwalior, MP, India; 2 Microbiology, Defence Research and Development Establishment, Jhansi road, Gwalior, MP, India; 3 Process Technology Development Division, Defence Research and Development Establishment, Jhansi road, Gwalior, MP, India

**Keywords:** Nerve agent, Soman, Acetylcholinesterase, HI-6 (1-[[[4-(aminocarbonyl)-pyridinio]-methoxy]-methyl]-2-[(hydroxyimino) methyl] pyridinium dichloride), Atropine and midazolam

## Abstract

**Background:**

Recent alleged attacks with nerve agent sarin on civilians in Syria indicate their potential threat to both civilian and military population. Acute nerve agent exposure can cause rapid death or leads to multiple and long term neurological effects. The biochemical changes that occur following nerve agent exposure needs to be elucidated to understand the mechanisms behind their long term neurological effects and to design better therapeutic drugs to block their multiple neurotoxic effects. In the present study, we intend to study the efficacy of antidotes comprising of HI-6 (1-[[[4-(aminocarbonyl)-pyridinio]-methoxy]-methyl]-2-[(hydroxyimino) methyl] pyridinium dichloride), atropine and midazolam on soman induced neurodegeneration and the expression of c-Fos, Calpain, and Bax levels in discrete rat brain areas.

**Results:**

Therapeutic regime consisting of HI-6 (50 mg/kg, i.m), atropine (10 mg/kg, i.m) and midazolam (5 mg/kg, i.m) protected animals against soman (2 × LD_50_, s.c) lethality completely at 2 h and 80% at 24 h. HI-6 treatment reactivated soman inhibited plasma and RBC cholinesterase up to 40%. Fluoro-Jade B (FJ-B) staining of neurodegenerative neurons showed that soman induced significant necrotic neuronal cell death, which was reduced by this antidotal treatment. Soman increased the expression of neuronal proteins including c-Fos, Bax and Calpain levels in the hippocampus, cerebral cortex and cerebellum regions of the brain. This therapeutic regime also reduced the soman induced Bax, Calpain expression levels to near control levels in the different brain regions studied, except a mild induction of c-Fos expression in the hippocampus.

**Conclusion:**

Rats that received antidotal treatment after soman exposure were protected from mortality and showed reduction in the soman induced expression of c-Fos, Bax and Calpain and necrosis. Results highlight the need for timely administration of better antidotes than standard therapy in order to prevent the molecular and biochemical changes and subsequent long term neurological effects induced by nerve agents.

## Background

Exposure to nerve agents such as sarin and soman causes an array of toxic effects, including hypersecretions, fasciculations, tremors, convulsions and death within minutes due to respiratory distress [1]. These toxic effects are mainly due to hyperactivity of the cholinergic system as a result of acetylcholinesterase (AChE) inhibition and the subsequent increase of the neurotransmitter acetylcholine at central and peripheral sites. The potential for exposure to nerve agents in a real world situation is likely to occur as a result of military operations, a terrorist incident, or accidental exposure, including demilitarization of weaponized material. Recent alleged use of nerve agent, sarin on civilian’s in Syria indicates their potential threat to civilian and military population. Use of chemical weapons (CW) still remains a major concern despite the efforts of the Organization for Prohibition of Chemical Weapons (OPCW, Netherlands, Nobel peace prize winner of 2013), to control the CW threat worldwide. In the event of nerve agent poisoning, an anticholinergic drug, such as atropine sulfate, is used to antagonize the effects of excess acetylcholine at muscarinic receptor sites, and an oxime, such as pralidoxime chloride (2-PAM-Cl), is used to reactivate any unaged inhibited enzyme [2,3]. However, this treatment regimen does not control the development of nerve agent-induced seizures [4,5]. Concomitant administration of an anticonvulsant drug such as diazepam is considered essential to optimize the regimen of carbamate pretreatment plus atropine and oxime therapy for severely exposed casualties [6,7]. Prolonged generalized seizures (status epilepticus) can begin rapidly after nerve agent exposure in humans [8-11]. Animal studies show these seizures can result in neuropathology and long-term behavioral deficits if not promptly controlled [12-16]. It is widely accepted that organophosphate (OP) induced seizure activities if not treated in a timely manner, can evolve into status epilepticus. This can cause an irreversible brain damage and long-term neurological, behavioral, and cognitive deficits [1,17]. The neuropathological consequences of OP-poisoning are related to the severity and duration of seizure activity [16,18].

Reactivation of inhibited acetylcholinesterase is considered to be an important element in post-exposure treatment. Bis-pyridinium oximes such as HI-6 can reactivate the phosphorylated enzyme if they are administered prior to the enzyme changes from the reactivatable to the unreactivatable state, the process referred to as “ageing” [3]. Diazepam (DZ), the preferred anticonvulsant benzodiazepine (BZ) for the treatment of OP-nerve agent-induced seizures and neuronal damage has been associated with unwanted effects, poor bioavailability, anticonvulsant tolerance, and dependence liability. Diazepam was also found to be not always completely effective in protecting animals against soman-induced neuropathology [19,20]. In a search for safer and more effective anticonvulsant BZs against OP-induced seizure and neuronal damage, Midazolam (MDZ), a non-selective and full positive allosteric modulator of GABA action at a variety of GABAA receptor subtypes [21], has recently been considered a possible anticonvulsant replacement for DZ [22]. The advantages that have been attributed to MDZ include its rapid bioavailability and the ease of administration by intranasal, sublingual, and intramuscular routes [22]. Gene expression studies during nerve agent exposure demonstrated several pathways in neurons including cholinergic, purinergic, NMDA-glutamatergic, GABAergic, catecholaminergic, serotogenic, calcium, and MAP kinase signaling along with genes related to ion channels, cytoskeletal proteins, cell adhesion, neurodegeneration, learning and memory, dementia/ataxia, mitochondrial dysfunction and apoptosis were altered significantly [23-26]. Excess muscarinic activation induced either by agonist application or by inhibition of AChE, results in long-lasting modifications of gene expression and protein levels of key cholinergic proteins [27,28]. Several studies have successfully employed c-Fos activity as a marker of neural activity [29] and increased c-Fos expression has been linked with organophosphate administration [30,31]. Convincing evidence suggest the role of free radicals in AChE inhibitors-induced neuronal cell and macromolecular damage [32,33].

The relative lethality of nerve agents as determined in animal studies is VX > Soman > Sarin > Tabun [34]. Among OP nerve agents, soman is considered as one of the most toxic due to its high lipophility and high affinity to the brain AChE and causes rapid ageing of AChE when compared to sarin, which is less lipophilic, however, its affinity to the brain AChE is also high [35,36]. Soman poisoning is also most difficult to counteract due to soman induced epileptic seizures and related brain damage, may resist to current therapies, if not treated immediately [19,20]. In view of this, soman represents most serious toxicant to test the therapeutic possibilities for nerve agents. Seizures might be one of the factors responsible for the late neurological effects of OP poisoning, it is important to determine the extent to which existing antidotes can reduce the seizures and diminish brain injury. Thus, there has been an active research effort to find out the molecular changes responsible for nerve agent-induced neurotoxicity to designing better drugs. A possible sequence of neurochemical events following nerve agent exposure is the inhibition of AChE causes an elevation of acetylcholine leading to massive activation of muscarinic and nicotinic receptors. Paralleled or sequenced activation of different pathways including phospholipases, protein kinases, proteases, transcription factors and generation of reactive oxygen and nitrogen species, may further account for the multiple neurotoxic effects resulting from nerve agent exposure. Thus, identifying the pathways and target genes will helps in the development of new pharmacological treatment to enhance recovery and repair processes in the nerve agent induced brain damage. In view of this, it is of interest to investigate the therapeutic effects of antidotes (HI-6, atropine and midazolam) on soman-induced neurodegeneration and the expression of c-Fos, Calpain, and Bax levels in the discrete rat brain areas.

## Methods

### Chemicals

Soman and HI-6 (1-[[[4-(aminocarbonyl)-pyridinio]-methoxy]-methyl]-2-[(hydroxyimino)-methyl] pyridinium dichloride) were obtained from Process Technology Development Division of DRDE, Gwalior. Purity of soman was greater than 98%, as verified by Gas chromatography. Mouse monoclonal antibodies for anti c-Fos (clone 2G9C3, Calbiochem,) anti μ-calpain and anti-β actin (clone, Ac-15), atropine sulphate and all other chemicals were obtained from Sigma chemicals Co. (St, Louis, U.S.A), unless otherwise mentioned. Luminal and Horseradish Peroxidase (HRP) substrate for chemiluminescence detection were purchased from Millipore Corporation, (Billerica, MA, USA). HRP conjugated anti mouse IgG was obtained from Dako Denmark A/S, DK2600 (Glostrup, Denmark).

### Drug treatment

Wistar male rats (100–120 g, 8–10 week old) were used in the present study. Rats were bred in Animal Facilities division of DRDE, Gwalior, India. All animal experimental procedures were approved by the Institutional Animals Ethics Committee (Registration No: 37/1999/CPCSEA, India). The care and maintenance of the animals were done as per the approved guidelines of the Committee for the Purpose of Control of the Experimental animals (CPCSEA, INDIA) under PCA acts 1960 and 1998. Animals were housed in vivarium under conditions of controlled room temperature between 21-25°C with 12 hr light–dark cycle. Food and water were provided ad libitum. Soman was dissolved in saline and administered to rats at 105 μg/kg (1 × LD_50_) as a single dose through subcutaneous (s.c) route and sacrificed at 30 min, 2.5 h, 1, and 7 day (n = 4 per each time point) time intervals. The sub cutaneous LD_50_ value of soman in the wistar rats was determined in our previous studies [37]. Vehicle (saline) control animals received an equivalent volume of 0.9% sodium chloride. In another set of experiments, animals were pretreated with the oxime, HI-6 (50 mg/kg, i.m), 30 min prior to challenge with 2 × LD_50_ soman (210 *μ*g/kg, s.c). One minute after soman exposure, animals were treated with atropine sulphate (10 mg/kg, i.m) followed by midazolam (5 mg/kg, i.m) on the onset of seizures and sacrificed at 30 min, 2.5 h, 1, and 7 day (n = 4 per each time point) time intervals. The rationale behind using the 2 × LD_50_ soman dose for soman plus antidotes treated animals is that previous studies has demonstrated that the LD_50_ value of nerve agents decreases with antidotal (atropine and HI-6) treatment [38]. In order to keep the number of experimental animals down, we have used 1 × LD_50_ dose to establish acute effects with soman alone as the death rate will be very high with 2 × LD_50_ dose of soman without any antidotes treatment. Control animals of this group were injected with antidotes comprising of HI-6, (50 mg/kg, i.m), 30 min prior to challenge with saline instead of soman. One minute later animals were treated with atropine sulphate (10 mg/kg, i.m) followed by midazolam (5 mg/kg, i.m) (*n* = 4; per each time point at 1 and 7 day). Rats were sacrificed by decapitation and brains were processed for further analysis. Samples were stored at −80°C until use.

### Assay of AChE activity (EC 3.1.1.7)

AChE activity was assayed according to the method of Ellman et al*.*[39], using acetylthiocholine iodide as the substrate. The enzyme activity was calculated as nmole substrate hydrolyzed min^−1^ ml^−1^ for plasma and nmole substrate hydrolyzed min^−1^ mg^−1^ protein for RBC and brain. Protein was estimated by Bradford method [40].

### Histopathological analyses of the brain after soman and antidotes treatment

Rats were anesthetized and subjected to transcardiacal perfusion with 0.9% saline (70 ml/min) until blood was cleared, followed by perfusion with 10% formalin at room temperature. Brains were immediately removed at 2 h, 1 and 7 day after soman, soman plus drugs and vehicle treated control rats and placed in fixative (10% formalin) for no longer than 48 h. Brains were then dehydrated and embedded in paraffin. Sections (10 μm thick) were cut and dried in an incubator at 37°C for 12 h before they were stained with neuron selective Fluoro-Jade B (FJ-B) as described [41]. After mounting, the tissue was examined using an epifluorescent microscope with blue (450–490 nm) excitation light and a filter for fluorescein isothiocyanate. Photomicrographs were taken with a digital microscope camera (AxioCam, Zeiss, Jena, Germany). For qualitative neuropathology evaluation of FJ-B stained sections, the following rating system initially described by McDonough et al. [42,43] and later applied for FJ-stained sections by Myhrer et al. [18], was used to score the extent of neuronal degeneration (neuropathology score) in each region is as follows, 0 = no neuropathology; 1 = minimal neuropathology (1–10% of the neuronal population was FJB-stained); 2 = mild neuropathology (11–25%); 3 = moderate neuropathology (26–45%); and 4 = severe neuropathology (>45%).

### RNA isolation and estimation

Total RNA was extracted from 50 mg of different regions of rat brain using RNeasy kit (Qiagen, Germany) following manufacturer’s protocol. The purity and quantity of total RNA was determined by measuring absorbance at A260/A280 ratios and then A260, respectively, using a UV-Spectrophotometer (BioTek, U.S.A). RNA having high purity ratio ranging from 1.9 to 2.1 was used for further real time PCR studies.

### Real-time RT-PCR

The quantitative real-time RT-PCR was carried out for the selected genes using gene specific primers from Quantitect primer assay kit (Qiagen, Germany). QuantiFast one step RT-PCR kit was used for real time PCR and RNA polymerase-II (RP-II) was used as an endogenous reference gene. Briefly, the reaction mixture consisted of 12.5 μl of 2 × QuantiFast SYBR Green RT-PCR Master Mix, 2.5 μl 10 × Quantitect primer mix, 0.25 μl of Quantifast RT Mix, 100 ng (2 μl) of template RNA and 8.75 μl nuclease free water in 25 μl reaction volume. The Roche Light cycler-480 system was used to monitor the SYBR Green signal at the end of each extension period for 40 cycles. The thermal profile consisted of 10 min of reverse transcription at 50°C for one cycle and 5 min of polymerase activation at 95°C, followed by 40 cycles of PCR at 95°C for 10 s, 60°C for 30 s for combined annealing/extension. The relative quantification levels in expression were determined using the 2nd derivative maximum analysis with the determination of the crossing points for each transcript. Crossing point values for each target gene were normalized to the respective crossing point values for the reference gene RP-II. Data are presented as normalized ratios of genes along with standard error using Roche Applied Science E-Method [44].

### Western analysis

Hippocampus, cerebellum and cerebral cortex tissues were homogenized in buffer containing 10 mM Tris, pH 7.6, 0.5 M sucrose, 1.5 mM MgCl_2_, 10 mM KCl, 10% glycerol, 1 mM EDTA, 1 mM dithiothreitol, and a protease inhibitor mixture (1 mM PMSF, 2 μg/ml aprotinin, leupeptin, and pepstatin A). The crude nuclear fraction was isolated by centrifugation at 4000x*g* for 5 min at 4°C. The nuclear pellet was resuspended in a lysis buffer containing 20 mM Tris (pH 7.6), 20% glycerol, 1.5 mM MgCl_2_, 0.2 mM EDTA, 0.3 M NaCl, 0.5 mM dithiothreitol, and 0.5 mM PMSF. Nuclear proteins were derived from the supernatant following centrifugation at 12,000x*g* for 20 min at 4°C. As for detecting calpain expression, brain tissues were homogenized in a tissue extraction buffer (Tris–HCl pH 6.8 containing 1 mM EGTA, 1 mM EDTA, and 1% Triton X-100), 20 mM β-glycerophosphate, 20 mM sodium fluoride, 1 mM sodium vanadate, and protease inhibitor cocktail (50 μl/g tissue). For the western analysis of calpain, the samples were centrifuged at 30,000×*g* for 30 min and resulting supernatants were used. After measuring protein concentrations using Bradford method [40], equal amounts of protein (40 μg) were diluted in 2x Laemmli’s buffer and subjected to 10% SDS-polyacrylamide gel electrophoresis. Proteins were transferred on to PVDF membranes and blocked with 5% non-fat dried milk dissolved in PBS (pH 7.5). Immunoblotting was carried out with anti-c-Fos antibody at 1:1000, 1:5000 dilutions for μ-calpain and β-actin overnight at 4°C. PVDF membranes were washed thrice in PBS containing 0.05% Tween 20 and incubated with anti-mouse horseradish peroxidase-conjugated secondary antibody at 1:3000 c-Fos, 1:8000 for μ-calpain and 1: 10,000 dilution for β-actin for 1 h at room temperature. Bands were developed by chemiluminescence detection using Luminol substrate. Immunoreactive bands of proteins were quantified as optical density (OD) by using Bio-Rad Quantity one software.

### Statistical analysis

Results were expressed as mean ± S.E and statistical analysis was performed with one-way analysis of variance (ANOVA), followed by Dunnett’s multiple comparison test using Sigma stat statistical software. A difference of p < 0.05 was considered significant.

## Results

### Clinical signs of nerve agent toxicity

Rats exhibited cholinergic signs of intoxication including tremors, chewing behavior, muscle fasciculations, salivation, respiratory distress and convulsions, within 5 to 15 min after soman (105 μg/kg, s.c.) administration. 40 to 50% animals that received 1 × LD_50_ dose of soman were died within 24 h after dosing (Table 1). Animals that received antidotal treatment (HI-6, atropine sulphate and midazolam) after soman (2 × LD_50_) exposure did not show severe classical cholinergic signs but were incapacitated up to 2 to 4 h with mild to moderate tremors and seizures. Thereafter animals were active and 15 to 20% of rats from these groups were died (Table 1) during the experimental period.

**Table 1:** Clinical toxicity of nerve agent exposure

**Treatment**	**Total number of animal used**	**Number of animals died**	**Number of animals survived**
Soman treated	60	28	32
Soman plus antidotes treated	36	6	30

### AChE activity in the blood and brain

**Figure 1:**
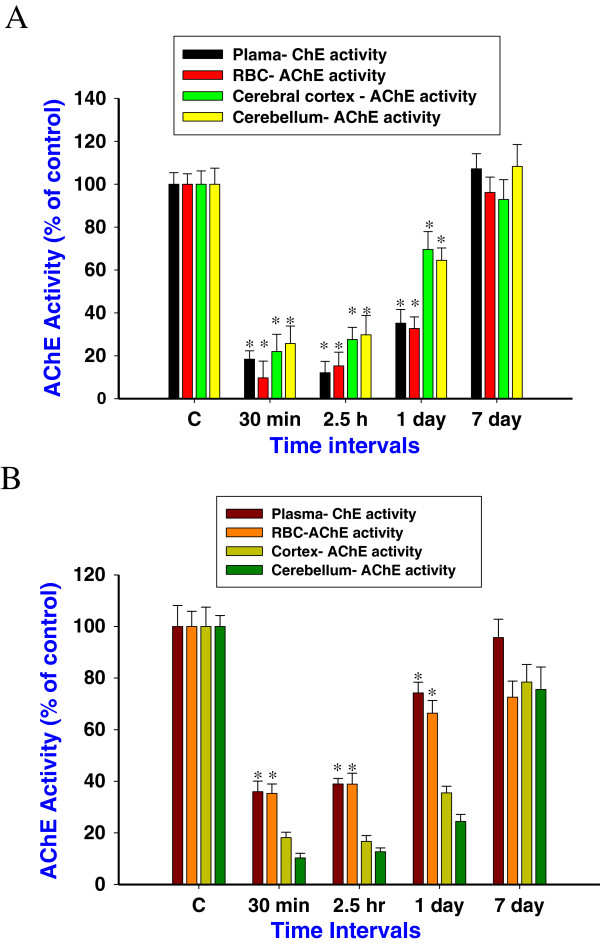
**Cholinesterase activity after soman and HI-6 treatment.** Cholinesterase activity in the rat plasma, RBC, cerebral cortex and cerebellum **(A)**, at 30 min, 2.5 h, 1, and 7 days after soman (105 μg/kg, s.c) exposure. Efficacy of HI-6 pretreatment (50 mg/kg, i.m, 30 min prior to soman exposure) on soman inhibited cholinesterase activity of rat plasma, RBC, cerebral cortex and cerebellum **(B)** at 30 min, 2.5 h, 1, and 7 day time points. AChE activity was presented as a percentage of control (n = 4 per each time point). *Significantly different from control. A difference of p < 0.05 was considered significant.

The effects of soman and HI-6 treatment on plasma, RBC and brain AChE activity were studied. Exposure to soman (105 μg/kg, s.c) reduced the cholinesterase activity to 17.4, 19.8, 32% and 9.7, 15 and 33.6% in the plasma and RBC (Figure 1A) at 30 min, 2.5 h and 1 day respectively. AChE activity in the cortex and cerebellum was reduced to 22, 24.6, 69.6% and 25.7, 29.8 and 64.5% at 30 min, 2.5 h and 1 day (Figure 1A), followed by the enzyme activity was recovered to near control level. HI-6 pretreatment reactivated the soman inhibited plasma ChE activity to 36, 42.4, 74% and RBC AChE activity to 35.3, 38.9 and 66.4% (Figure 1B), to control level at 30 min, 2.5 h and 1 day time points. Thereafter AChE enzyme activity was restored to near control levels. No significant reactivation in brain AChE activities were observed after HI-6 treatment (Figure 1B), when compared to soman exposed group, indicate that HI-6 cannot reactivate soman inhibited CNS AChE.

### Neuropathology of brain regions after nerve agent and antidotes treatment

**Figure 2:**
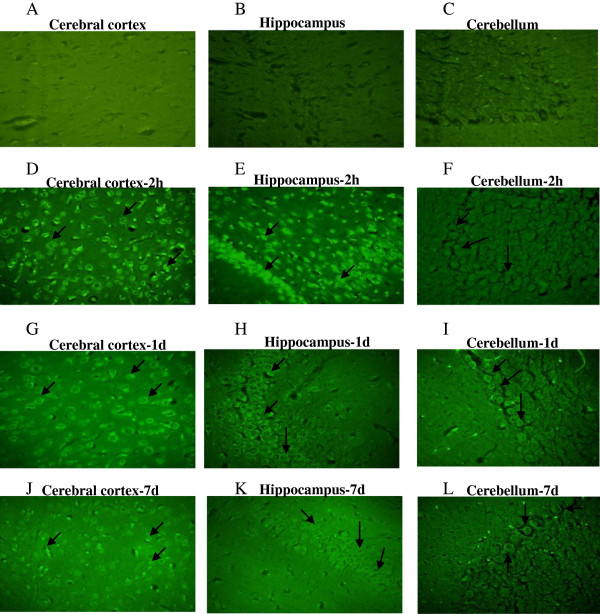
**Neurodegeneration after soman poisoning.** Soman induced neurodegeneration was detected using neuron selective Fluoro-Jade-B staining: Representative photomicrographs of Fluoro-Jade-B positive neurons of cerebral cortex, hippocampus (CA1 sub region), and cerebellar regions obtained from vehicle **(A, B and C)** and soman (105 μg/kg, s.c) treated rats sacrificed at 2 h **(D, E and F)**, 1 day **(G, H and I)** and 7 day **(J, K and L)** time points (n = 4 per each time point) taken with 100 magnifications.

**Figure 3:**
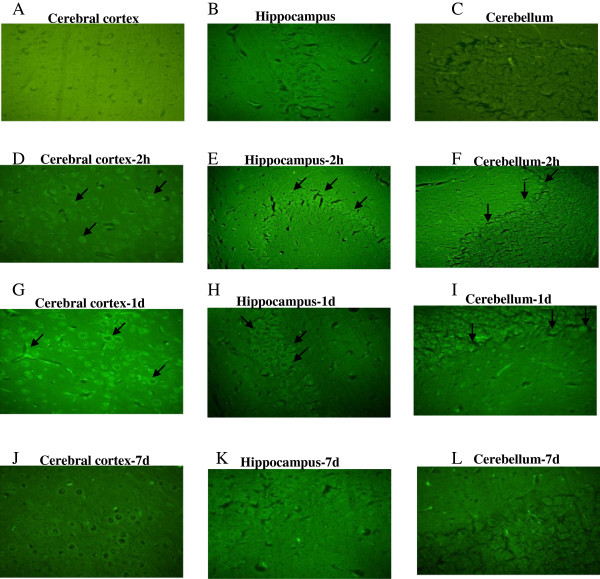
**Efficacy of antidotes on soman induced neurodegeneration.** Efficacy of antidotes (HI-6 (50 mg/kg, i.m), atropine (10 mg/kg, i.m) and midazolam (5 mg/kg, i.m) on soman induced neurodegeneration: Representative photomicrographs of Fluoro-Jade B stained cerebral cortex, hippocampus (CA1 sub region) and cerebellum regions obtained from vehicle **(A, B and C)** and after antidotes + soman treatment animals sacrificed at 2 h **(D, E and F)**, 1 day **(G, H and I)**, 7 day **(J, K and L)** time points (n = 4 per each time point) taken with 100 magnifications.

Neuron selective Fluoro-Jade-B staining was used to identify degenerating neurons in different brain regions of soman exposed rats. FJ-B, an anionic fluorescein derivative that binds with high affinity to degenerating neurons that presumably expresses a strong basic molecule. In the present study, Fluoro-Jade positive neurons in the brain sections of soman (1 × LD_50_) challenged rats and rats that received antidotes (HI-6, atropine sulphate and midazolam) were studied at 2 h, 1 and 7 day after treatment. This staining procedure is a sensitive and reliable marker for neuronal degeneration that results from OP-poisoning and traumatic brain injury [41]. Large numbers of shrunken FJ-B positive neurons were consistently seen in the hippocampus (CA1 sub region), cerebral cortex and in the cerebellum regions (Figure 2) of animals that survived the exposure to 1 × LD_50_ soman. The number of degenerating neurons increased with the severity of the acute signs of intoxication following the soman exposure as maximum necrotic neurons were visible at 2 hours and 1 day, particularly in the hippocampus (CA1 sub region), and cerebral cortex indicate their sensitivity when compared least sensitive cerebellum region of the brain (Figure 2). Treatment with antidotes (HI-6, atropine sulphate and midazolam) after soman exposure prevented soman-induced neurodegeneration as FJ-B positive neurons were reduced in all the brain regions obtained from animals that received antidotes (Figure 3), except very few necrotic neurons were observed in the sensitive regions such as hippocampus (CA1) and cerebral cortex.

### Efficacy of HI-6, atropine and midazolam on soman induced c-Fos and Bax expression

**Figure 4:**
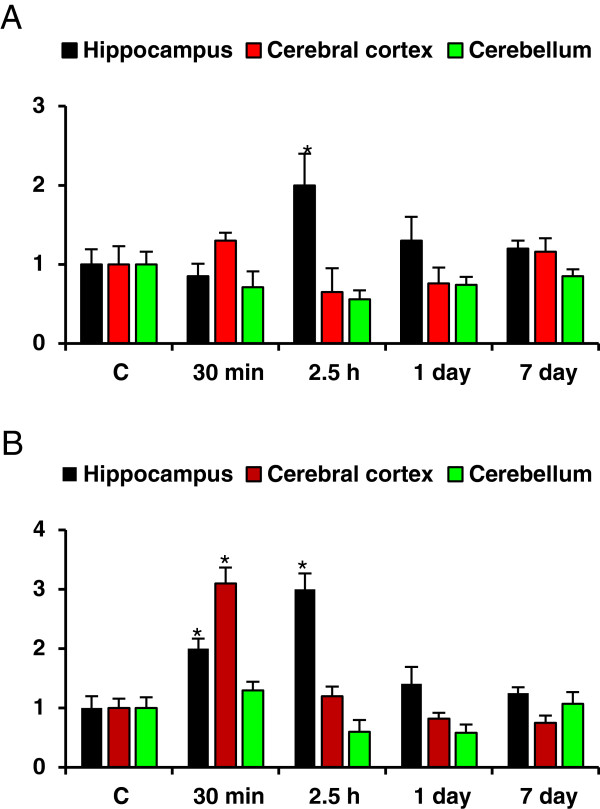
**Efficacy of antidotes on soman induced c-Fos and Bax mRNA levels**. Effect of HI-6 (50 mg/kg, i.m), atropine (10 mg/kg, i.m) and midazolam (5 mg/kg, i.m) on soman induced Bax (Figure 4A) c-Fos (Figure 4B) mRNA levels of rats sacrificed at 30 min, 2.5 h, 1d and 7d time points (n = 4 per each time point). Quantitative real-time RT-PCR was carried out and results were presented as fold change over control (presented as 1.0 fold) after normalizing the data (n = 4 per each time point) with housekeeping gene, RNA polymerase-II (RP-II), which did not show any significant changes in the mRNA expression among control and experimental groups (Data not presented). *Significantly different from control group. A difference of p < 0.05 was considered significant.

**Figure 5:**
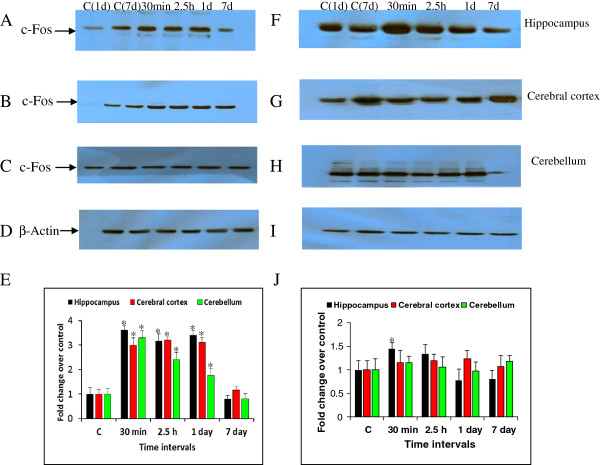
**c-Fos protein levels after soman and antidotes treatment.** Efficacy of HI-6 (50 mg/kg, i.m), atropine (10 mg/kg, i.m) and midazolam (5 mg/kg, i.m) on soman induced c-Fos protein levels of rats sacrificed at 30 min, 2.5 h, 1d and 7d time points (n = 4 per each time point). c-Fos immunoreactivity levels in the rat hippocampus **(A)**, cerebral cortex **(B)** and cerebellum **(C)** after soman exposure (E-bar graph). c-Fos immunoreactivity levels in the rat hippocampus **(F)**, cerebral cortex **(G)** and cerebellum **(H)** after antidotes treatment (**J**- bar graph). β-actin **(D and I)** was used as protein loading control. Letters on blot C1 (1d) and C2 (7d) indicate the control samples collected after 1 and 7 days after HI-6 (50 mg/kg, i.m), saline (100–120 μl/rat, s.c), atropine sulphate (10 mg/kg, i.m), and midazolam (5 mg/kg, i.m), treatment. Densitometric quantification of band intensities are presented as fold change over control (presented as 1.0). A difference of p < 0.05 was considered significant (*).

Animals that received the antidotes comprising of HI-6, 30 min prior to soman (210 μg/kg, s.c) exposure followed by atropine and midazolam (1 and 10 min after soman challenge) has reduced soman induced Bax mRNA levels (Table 2), except one fold induction in the hippocampus region at 2.5 hour (Figure 4A) time point. Similarly, these rats displayed low mRNA expression levels of c-fos mRNA in the hippocampus, cortex, and cerebellum (Figure 4B), when compared to soman exposed animals (Table 2), except a moderate increase at early time points (30 min and 2.5 h). Protein levels of c-Fos were increased significantly by 3.62, 3.17, 3.42 fold in the hippocampus (Figure 5A), 3.0, 3.2, 3.12 fold in the cerebral cortex (Figure 5B) and 3.3, 2.42, 1.76 fold in the cerebellum (Figure 5C) at 30 min, 2.5 h and 1 day after soman exposure (Figure 5E). Immunoreactivity levels of c-Fos was restored to near control levels in the cerebral cortex (Figure 5G) and cerebellum (Figure 5H), while there was a mild to moderate induction at very early time points (30 min and 2.5 h) in the hippocampus (Figure 5F) after treatment with antidotes (Figure 5J). β-actin (Figure 5D and I) was used as protein loading control.

**Table 2:** Effect of soman (105 μg/kg) on c-Fos and Bax mRNA levels in different brain regions

**Time points**	**Fold change over control**					
	**Hippocampus**		**Cerebral cortex**		**Cerebellum**	
**Gene name**	c-Fos	Bax	c-Fos	Bax	c-Fos	Bax
Control animals	1.0±0.18	1.0±0.21	1.0±0.24	1.0 ±0.18	1.0 ±0.16	1.0±0.13
***Soman exposed***						
30 min	3.5*±0.47	2.6*±0.13	5.0* ±0.8	2.5*±0.24	3.0*±0.4	0.72±0.24
2.5 h	4.2*±0.18	1.6*±0.31	3.1*±0.71	2.0*±0.3	1.8*±0.25	0.75**±**0.14
1 day	3.4*±0.4	1.8*±0.27	3.8*±0.8	1.6*±0.18	1.5*±0.19	0.45*±0.18
7 day	0.65**±**0.3	1.5*±0.18	0.78**±**0.23	1.25±0.25	0.8±0.18	0.52*±0.09

Quantitative real-time RT-PCR was carried out and results are presented as fold change over control (presented as 1.0 fold) after normalizing the data (n=4 per each time point) with housekeeping gene, RNA polymerase-II (RP-II), which did not show any significant changes in the mRNA expression among control and experimental groups (Data not presented). *Significantly different from control group. A difference of p< 0.05 was considered significant.

### Expression of μ-calpain in the rat brain after soman and antidotes treatment

**Figure 6:**
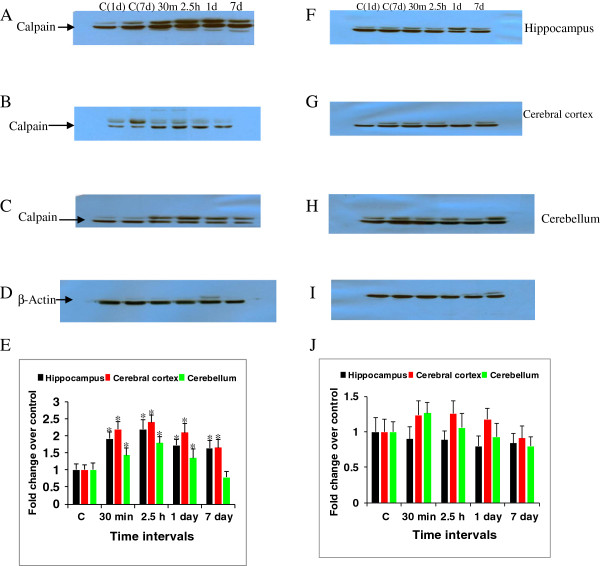
**Calpain expression after soman and antidotes treatment.** Efficacy of HI-6, atropine and midazolam on soman induced calpain protein levels of rats sacrificed at 30 min, 2.5 h, 1d and 7d time points (n = 4 per each time point). Calpain immunoreactivity levels in the rat hippocampus **(A)**, cerebral cortex **(B)** and cerebellum **(C)** after soman poisoning (**E**- bar graph). Calpain levels in the rat hippocampus **(F)**, cerebral cortex **(G)** and cerebellum **(H)** after antidotes treatment (**J**-bar graph). β-actin **(D and I)**, was used as protein loading control. A difference of p < 0.05 was considered significant (*).

Expression of μ-calpain was studied in the rat brain following soman exposure. The immunoreactivity levels of μ-calpain increased significantly by 1.92, 2.2, 1.73, 1.64 fold in the hippocampus (Figure 6A), 2.2, 2.4, 2.1,1.65 fold in the cerebral cortex (Figure 6B) and 1.43, 1.8, 1.35, 0.78 fold in the cerebellum (Figure 6C) at 30 min, 2.5 h, 1 and 7 day post soman exposure time points (Figure 6E). While, μ-calpain levels in the hippocampus (Figure 6F), cerebral cortex (Figure 6G) and cerebellum (Figure 6H) of rats that received antidotes (Figure 6J) were reduced to near control levels when compared to soman treated rats. β-actin (Figure 6D and I) was used as protein loading control.

## Discussion

Exposure to nerve agents can cause long term neurological effects via a complex and diverse mechanisms. Poor understanding of molecular and biochemical changes that cause persistent neurological impairments after nerve agent exposure is hampering the development of complete therapy against nerve agents. Several studies that used diazepam as an OP treatment found that it could block the seizures and reduce neuropathology when administrated in combination with other therapies [42]. However, protection against brain damage is not complete with diazepam. The bispyridinium oxime, HI-6, together with atropine and potent anticonvulsant such as midazolam treatment, can be effective therapy for blocking soman induced molecular changes and brain damage. The present study was designed to evaluate the efficacy of HI-6, atropine and midazolam on soman induced molecular and biochemical changes in the discrete rat brain areas. Different parameters that were addressed in the study include: the effects of antidotes on nerve agent induced c-Fos, Calpain, and Bax expression levels and neurodegeneration in the different rat brain regions.

Neuron selective FJ-B staining was used to detect neurons undergoing degeneration in the brain sections of soman exposed rats that received either no treatment or antidotes comprised of (HI-6, atropine sulphate and midazolam). Results showed that soman induced neurodegeneration can be significantly reduced by timely administration of therapeutic regime consisting of potent anticonvulsant, midazolam. Neurodegeneration in the hippocampus, cortex, and amygdala is a hallmark of nonfatal OP exposure in animals. It probably contributes to the delayed cognitive effects observed in the survivors of the sarin subway attack in Japan [45]. The intensity and duration of OP-induced seizures appear to be major determinants of the degree of neurodegeneration in the brains of OP-intoxicated rodents and nonhuman primates [13,20]. Cholinergic hyperactivity initiates nerve agent induced seizures and triggers glutamatergic hyperactivity, which sustains and reinforces seizures and is eventually responsible for excitotoxic damage in the several parts of cortex, amygdala, and hippocampus [5,17,46]. It is crucial to control soman-induced seizures at an early stage (<20 min) to avoid brain damage and cognitive dysfunctions [18]. Intoxication of rodents and nonhuman primates with soman and other OP compounds triggers neuronal loss in the brain, especially in the pyriform cortex, amygdala, and in the hippocampus [47].

We have recently shown that c-fos and Bax expression was significantly increased in the rat brain after soman exposure [31]. The transient immediate elevation of c-Fos may lead to the activation of other genes that are involved in the expression of proteins implicated in the development of delayed neurotoxic effects of nerve agents [31,48,49]. Recent reports demonstrated that role of OP induced acetylcholine and seizures in the c-Fos induction [50,51]. In the present study, when these drugs were given immediately after soman exposure has shown promising protection against mortality and brought down the Bax gene expression to near control levels. Soman induced c-Fos levels were also significantly reduced, except moderate induction at early time points. c-Fos induction can be a sensitive indicator of soman induced neuronal activation such as seizures, and early c-Fos induction even after antidotal treatment indicate the involvement and activation of many molecular and signaling pathways immediately after nerve agent exposure. It is hypothesized that initial cholinergic neurotransmission, followed by non-cholinergic neurotransmission persisting for hours after the initial exposure of nerve agents, results in cognitive and motor deficits in animals [20]. The immunoreactivity levels of soman induced μ-calpain in the rat brain areas were reduced by antidotal treatment used in the present study. Calpains constitute a family of cysteine proteases that are activated by calcium at neutral p^H^. Calpains activation under physiological conditions is critical for normal synaptic function and memory formation in the CNS and hyper activation under pathological conditions that involve sustained calcium overload, generally associated with severe cellular challenge or damage [52,53]. Increasing evidence suggest that AChE inhibitors-induced neuronal damage is a consequence of a series of extra and intracellular events leading to the intracellular accumulation of Calcium and the generation of oxygen-derived free radicals. These, in turn, cause irreversible damage to cellular components [32,33].

## Conclusion

To summarize the present study findings, results presented herein suggest that therapeutic treatment comprising of HI-6, atropine sulphate and midazolam has significantly protected animals from death and reduced the soman induced biochemical changes including neuronal cell death, expression of Bax, Calpain and c-Fos in the cerebral cortex, hippocampus and cerebellum. The early response after nerve agent exposure can be attributed to AChE inhibition, increasing acetylcholine levels, and corresponding induction of signal transduction processes [26,54,55]. Several organophosphates, including sarin, can exert direct effects on multiple brain proteins besides their effects mediated through AChE inhibition [56,57]. Our findings might provide an additional understanding of diverse targets of OPs in the brain. Results suggest that in order to prevent the nerve agent induced molecular and biochemical changes, better antidotes should be administered immediately as the existing drugs were unable to completely reverse the soman induced molecular changes. This is particularly important in the case of acute exposures such as terrorist attacks or in the military operations, so that drugs will prevent the long term changes of the nerve agents, initially in the most acute phase.

## Abbreviations

HI-6: (1-[[[4-(aminocarbonyl)-pyridinio]-methoxy]-methyl]-2-[(hydroxyimino) methyl] pyridinium dichloride); i.m: Intramuscular; s.c: Subcutaneous; FJ-B: Fluoro-Jade B; AChE: Acetylcholinesterase; CWA: Chemical weapons; OPCW: The organization for prohibition of chemical weapons; 2-PAM-Cl: Pralidoxime chloride; OP: Organophosphate; DZ: Diazepam; BZ: Benzodiazepine; MDZ: Midazolam; CNS: Central nervous system; CPCSEA INDIA: The committee for the purpose of control of the experimental animals; DNPH: Dinitrophenylhydrazone; ANOVA: Analysis of variance.

## Competing interests

The authors declare that there are no financial, non-financial competing interests or personal conflicts of interest in this publication.

## Authors’ contributions

GRR and BKB conceived and designed the experiments. GRR injected the animals with drugs and soman, collected the biological samples and performed the experiments and analyzed the data. JA has synthesized the soman and HI-6. PA analyzed the real-time QPCR data. GRR wrote the paper. All authors critically revised the manuscript and approved the final submitted version.

## Authors’ information

GRR and BKB are affiliated to Biochemistry division, PA is affiliated to Microbiology division, and JA is affiliated to Process Technology Development Division of Defence Research and Development Establishment, Jhansi road, Gwalior, M.P. India.
